# Prognosis Research Strategy (PROGRESS) 2: Prognostic Factor Research

**DOI:** 10.1371/journal.pmed.1001380

**Published:** 2013-02-05

**Authors:** Richard D. Riley, Jill A. Hayden, Ewout W. Steyerberg, Karel G. M. Moons, Keith Abrams, Panayiotis A. Kyzas, Núria Malats, Andrew Briggs, Sara Schroter, Douglas G. Altman, Harry Hemingway, for the PROGRESS Group

**Affiliations:** 1 School of Health and Population Sciences, University of Birmingham, Birmingham, United Kingdom; 2 Department of Community Health and Epidemiology, Dalhousie University, Halifax, Nova Scotia, Canada; 3 Department of Public Health, Erasmus MC, Rotterdam, Netherlands; 4 Julius Center for Health Sciences and Primary Care, UMC Utrecht, Utrecht, Netherlands; 5 Centre for Biostatistics & Genetic Epidemiology, Department of Health Sciences, School of Medicine, University of Leicester, Leicester, United Kingdom; 6 Department of Oral and Maxillofacial Surgery, North Manchester General Hospital, Pennine Acute NHS Trust, Manchester, United Kingdom; 7 Spanish National Cancer Research Centre (CNIO), Madrid, Spain; 8 Health Economics & Health Technology Assessment, Centre for Population & Health Sciences, University of Glasgow, Glasgow, United Kingdom; 9 BMJ, BMA House, Tavistock Square, London, United Kingdom; 10 Centre for Statistics in Medicine, University of Oxford, Oxford, United Kingdom; 11 Department of Epidemiology and Public Health, University College London, London, United Kingdom

## Abstract

In the second article in the PROGRESS series on prognostic factor research, Sara Schroter and colleagues discuss the role of prognostic factors in current clinical practice, randomised trials, and developing new interventions, and explain why and how prognostic factor research should be improved.

**Summary Points**

- The PROGRESS series (http://www.progress-partnership.org) sets out a framework of four interlinked prognosis research themes and provides examples from several disease fields to show why evidence from prognosis research is crucial to inform all points in the translation of biomedical and health related research into better patient outcomes. Recommendations are made in each of the four papers to improve current research standards.
- What is prognosis research? Prognosis research seeks to understand and improve future outcomes in people with a given disease or health condition. However, there is increasing evidence that prognosis research standards need to be improved.
- Why is prognosis research important? More people now live with disease and conditions that impair health than at any other time in history; prognosis research provides crucial evidence for translating findings from the laboratory to humans, and from clinical research to clinical practice.
- A prognostic factor is any measure that, among people with a given startpoint (such as diagnosis of disease), is associated with a subsequent endpoint (such as death).
- Prognostic factors have many potential uses: for example, they help define disease at diagnosis, inform clinical and therapeutic decisions (either directly or as part of prognostic models for individualised risk prediction), enhance the design and analysis of intervention trials, and help identify targets for new interventions that aim to modify the course of a disease or health condition.
- Limitations in current prognostic factor research include publication bias, reporting biases, poor statistical analyses, and inadequate replication of initial findings.
- To address these issues we recommend that large, prospective, registered, and protocol supported prognostic factor studies are needed with suitable sample size, appropriate statistical analyses, and transparent reporting of all factors and outcomes considered. Initial exploratory studies are also important, but must be labelled as such.
- A factor's prognostic ability should be examined across multiple studies, and we recommend increased use of (ideally prospectively planned) meta-analysis of individual participant data, as it potentially alleviates any reporting biases and analysis deficiencies in primary studies.
- For each factor identified as prognostic, there should be greater understanding of how it can be used to improve clinical outcomes, including whether it is useful within the clinical management of patients and whether it informs the development of novel interventions.
- The other papers in the series are:

○ PROGRESS 1: *BMJ* 2013, doi:10.1136/bmj.e5595
○ PROGRESS 3: *PLOS Med* 2013, doi:10.1371/journal.pmed.1001381
○ PROGRESS 4: *BMJ* 2013, doi:10.1136/bmj.e5793

*Prognostic factor research aims to identify factors associated with subsequent clinical outcome in people with a particular disease or health condition. In this article, the second in the PROGRESS series, the authors discuss the role of prognostic factors in current clinical practice, randomised trials, and developing new interventions, and explain why and how prognostic factor research should be improved.*

A prognostic factor is any measure that, among people with a given health condition (that is, a startpoint), is associated with a subsequent clinical outcome (an endpoint). For example, in many cancers tumour grade at the time of histological diagnosis is a prognostic factor because it is associated with time to disease recurrence or death. This is illustrated in Figure 1, which shows that in 246 patients with breast cancer treated with tamoxifen the survival times were shorter in those with a higher tumour grade status [1]. Prognostic factors thus distinguish groups of people with a different average prognosis and thus inform and enhance the basic prognosis summaries that were discussed for outcomes research in paper 1 of our series [2].

**Figure 1:**
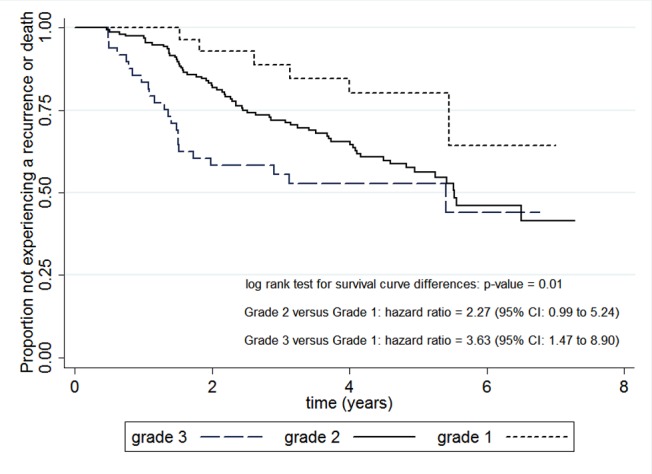
Tumour grade as a prognostic factor in breast cancer. Kaplan-Meier curves for disease-free survival for three groups of breast cancer patients defined by tumour grade status (1, 2, or 3). Curves are derived from 246 breast cancer patients treated with tamoxifen who had 94 recurrences or deaths over a possible 7 years of follow-up (reproduced with published data relating to Schumacher et al [1]). The distinct curves, significant log rank result, and hazard ratio estimates suggests tumour grade is a prognostic factor, as it identifies three groups of patients with a different average prognosis.

In many diseases, the most researched prognostic factors are biomarkers [3]. Biomarkers include a diverse range of biological (including genomic [4], transcriptomic [5], proteomic, metabolomic), pathological, imaging, clinical, and physiological variables: for example, in children with neuroblastoma, elevated expression of the MYCN oncogene is associated with a shorter time to recurrence and death [6]. Symptoms and behavioural and psychosocial characteristics may also be prognostic: for example, in patients with low back pain, psychosocial factors such as maladaptive pain coping and comorbid depression and higher levels of functional limitation at clinical presentation have been shown to be associated with worse outcomes [7].

Prognostic factors may also be measured outside the individual, at an ecological level (in which the exposure of individuals is inferred), such as area-level social deprivation, healthcare access and quality, and physical environment. For example, mortality and morbidity rates in UK patients with coronary heart disease vary by socioeconomic group (rates are higher in lower socioeconomic groups) and by geographical area (rates are highest in Wales, North West England, and the Northern England and Yorkshire regions and lowest in South East England) [8].

Prognostic factor research aims to discover and evaluate factors that might be useful as modifiable targets for interventions to improve outcomes, building blocks for prognostic models, or predictors of differential treatment response. Prognostic factor research is found extensively in the medical literature, with thousands of studies published each year [9]. Genuine prognostic factors can play an important role in many of the pathways towards improved clinical outcomes (see pathways schema at the bottom of Figure 2, introduced in paper 1 in our PROGRESS series [2]). The first aim of this article is to illustrate the broad potential of identifying prognostic factors, from their use within current clinical practice to their implications for randomised trial design and developing new interventions. The second aim is to highlight why the overall quality of prognostic factor research is currently poor [3] [10]–[12] and to make recommendations for improving the reliability of the accumulated prognostic factor evidence over time in order to ensure identification of factors that can be used to influence practice.

**Figure 2:**
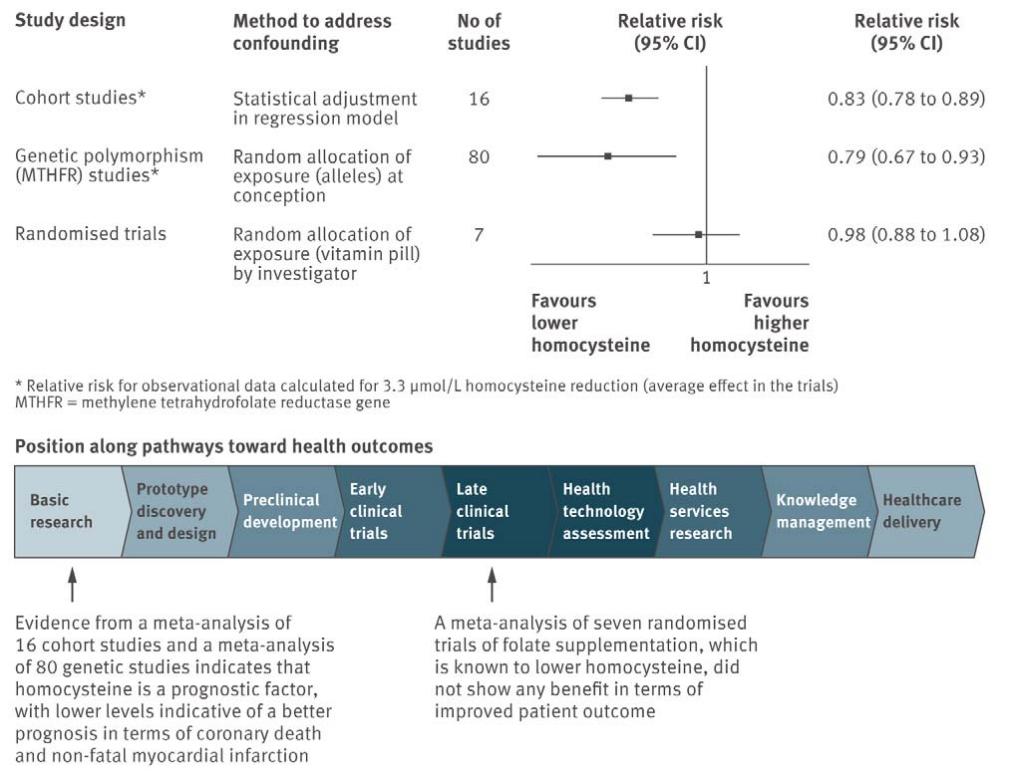
Evaluation of whether homocysteine is a prognostic factor, and whether modifying it improves clinical outcome in patients with coronary disease (drawn from data in [35]). Path element adapted from Chart 7.1 in the Cooksey report (2006) http://bit.ly/Ro27rL (made available for use and re-use through the Open Government License).

## Importance of Prognostic Factors for Current Clinical Practice

Evidence from prognostic factor research has a broad array of uses in healthcare and clinical research. We start by considering how they are currently used to influence clinical decision making.

### Changing How Diseases and Health Conditions Are Defined

A fundamental role of prognostic factors is to aid the definition of a disease or health condition [13], to inform or refine diagnosis, and to enhance average prognosis summaries [2]. For example, diagnosis of cancer is usually accompanied by the stage of disease, which is based on the prognostic factors of tumour size, nodal status, and metastasis. Another example is CD4 cell count; this was not initially included in the definition of AIDS, but evidence that it was a strong prognostic factor (associated with a range of measures of disease progression) and understanding of its biological significance led to its inclusion in diagnostic criteria. Future developments are anticipated from the use of genomics to discover new “classes” of disease, and prognosis research is evaluating gene expression signatures [14].

### Informing Treatment Recommendations and Individual Patient Management

Randomised controlled trials are the main study design for informing treatment decisions. However evidence on individual prognostic factors may be used to further inform treatment choices. For example, the use of drug eluting stents for the treatment of coronary artery disease was restricted by the National Institute for Health and Clinical Excellence (NICE) to patients with coronary artery lesions longer than 15 mm, a prognostic factor for the probability of restenosis [8]. Patients with such lesions had a worse prognosis and thus were considered to have a greater potential to gain from receiving drug eluting stents than patients without it.

The identification of new prognostic factors may also widen the criteria for patients suitable for treatment. For example, a recent study of women undergoing sentinel node biopsy for breast cancer indicates that, even after adjustment for established prognostic factors (age, tumour size, tumour grade, and hormone receptor status), the presence of isolated tumour cells is associated with a higher rate of recurrence and death, and is thus a prognostic factor [15]. The American Joint Committee on Cancer currently defines patients with isolated tumour cells as “node negative”; the authors of this study conclude that this should be re-evaluated, potentially expanding the group of patients in whom adjuvant therapy is currently given [15].

### Building Blocks for Prognostic Models

Individual risk prediction is usually poor when based on just one factor. To improve the targeting of interventions to patients based on their predicted individual risk of subsequent outcomes, decision makers can use multiple prognostic factors combined within a prognostic model. For example, a prognostic model developed to help identify patients with traumatic brain injury who are likely to have an unfavourable six month outcome [16] involves the prognostic factors of age, motor score, pupillary reactivity, computed tomographic characteristics, and laboratory parameters. Some prognostic models for predicting individual outcome risk are being used in clinical practice, such as the GRACE score in acute myocardial infarction or the ADJUVANT! score in breast and other cancers, and we consider them further in the third paper in our series [17].

### Potential Predictors Of Treatment Response for Stratified Medicine

The current drive toward stratified medicine requires the identification of factors associated with more benefit or less harm from a specific treatment [18]. Prognostic factors (or prognostic models for individual risk prediction based on multiple prognostic factors) are natural variables to consider for this role; not only do they identify those at highest risk, who generally benefit most, but they may even predict treatment response. An example of a prognostic factor that also predicts treatment response is epidermal growth factor receptor tyrosine kinase (EGFR-TK) status in non-small cell lung cancer. NICE has recommended use of gefitinib as first line treatment for this disease only in those patients who tested positive for EGFR-TK [19]. We note, though, that only a few prognostic factors will also be predictive of differential treatment response [20]. We return to stratified medicine in our fourth PROGRESS paper [21].

### Use for Monitoring Disease Progression

Clinicians use prognostic factors to monitor changes in disease status and treatment response over time [22]. For example, measurement of haemoglobin A_1c_ (HbA_1c_) levels in people with diabetes [23] allows clinicians, with one blood test, to assess the average serum glucose values over the previous 120 days and to make inferences about how well interventions have controlled glucose levels. Evidence that HbA_1c_ is a prognostic factor (strongly associated with the risk of subsequent vascular events) influenced guideline recommendations that it should be routinely assessed [24]. Other examples of prognostic factors being used for monitoring include CD4 count in HIV infection, blood pressure or temperature in critical care medicine, and carcinoembryonic antigen (CEA) levels in colorectal cancer [25].

## Importance of Prognostic Factors for Intervention Research and Trials

Alongside their usefulness in clinical practice, prognostic factors also inform and facilitate intervention research.

### Development of New Interventions to Modify a Prognostic Factor

Prognostic factors may suggest the development of new interventions, or new applications of existing interventions, under the assumption of a causal relationship between the factor and subsequent outcome. A causal factor is prognostic because it directly or indirectly causes future outcomes, and so modifying a causal prognostic factor will change the average disease course. An example of a prognostic factor that has subsequently informed intervention strategies in the management of low back pain is the psychological behavioural factor “fear avoidance beliefs.” This factor describes exaggerated pain perceptions and fear of experiencing pain leading to avoidance of activities that are perceived to cause pain. Evidence supporting an association between fear avoidance beliefs during an acute episode of low back pain and subsequent chronic disability includes several prospective cohort studies, synthesised in systematic reviews [26],[27]. Clinicians and researchers have hypothesised that fear avoidance beliefs may be a modifiable prognostic factor and have recommended patient management to decrease fear avoidance and promote normal activities (such as through graded activity exposure) [28],[29]. A randomised controlled trial in primary care evaluated use of fear reducing and activating techniques and found a decrease in disability related to low back pain [30]. There are many other examples of prognostic factors hypothesised to be modifiable and which could stimulate new interventions targeted to modify them. For example, mild anaemia is prognostic in stable coronary artery disease [31], and blood glucose measured at admission is prognostic in traumatic brain injury [32],[33].

It should never be assumed that intervening on a prognostic factor will improve outcome. Before embarking on a randomised trial to evaluate the benefit of modifying a prognostic factor, it is important to recognise that most prognostic factors will not be causal and are merely associated with the true (often unknown) causal factors. Indeed, as in aetiological research, it is difficult to establish whether a particular factor is truly causal, and one must consider multiple sources of evidence from high quality studies [34]. For example, is there repeated confirmation (from multiple studies) that the factor is prognostic? Does the factor retain prognostic value even after adjustment for other prognostic factors? Is there evidence of how the factor fits on the (causal) pathway from disease to outcome, and an understanding of the biological mechanism involved? Do randomised trials of interventions that modify the prognostic factor provide evidence of improved outcome?

The example of homocysteine in coronary artery disease illustrates the issues of modification and causality of a prognostic factor (Figure 2) and the role of three different study designs [35]. A meta-analysis of 16 observational cohort studies suggested that, after adjustment for other prognostic factors (confounders), lower homocysteine levels are associated with a better prognosis in terms of coronary death and non-fatal myocardial infarction. This evidence is concordant with that from a meta-analysis of 80 genetic studies that used a Mendelian randomisation design (where the variants associated with homocysteine were randomly allocated at conception) to adjust for confounders. These two study designs suggest that homocysteine is a prognostic factor and provide a rationale for experimental studies lowering homocysteine. However, a meta-analysis of seven randomised trials of folate supplementation, which is known to lower homocysteine, did not show any benefit in terms of improved patient outcome. This trial evidence is consistent with different interpretations, including that homocysteine is not causal (as modifying it did not improve outcome) and that homocysteine is causal but the lack of reversibility was due to the particular intervention used. Thus, even in situations with a large evidence base of how modifying the factor changes outcome, inferences that a prognostic factor is causal are problematic and should be treated with caution.

### Aiding Design and Analysis of Intervention Studies

Prognostic factors can be important in the design and analysis of intervention studies, including randomised trials [36] where stratified randomisation (or minimisation) may be used to ensure treatment groups are balanced across levels of a prognostic factor. If prognostic factor values are not balanced across treatment groups of interest then they may mask the true effect of an intervention on disease outcome. In other words, prognostic factors are potential confounding factors, and so in cohort studies or trials with unbalanced treatment groups it may be desirable to adjust for them in the statistical analysis to limit or reduce potential confounding. For example, Royston et al show how adjustment (within a statistical model) for an established prognostic factor in randomised trials with baseline imbalance can change the inference about treatment effectiveness [37]. Even in randomised trials or genome-wide studies with no baseline imbalance, statistical analyses may adjust for prognostic factors to gain power [38]–[40].

## Prognostic Factor Research: From Discovery to Replication

Given these broad potential uses of prognostic factors, high quality research to identify prognostic factors is essential. To this end, different phases of such research have been noted [34],[41]. Broadly, prognostic factor evidence should evolve from initial studies that aim to identify or explore factors about which little if anything is known in relation to prognosis (exploration), to studies that seek to evaluate previously identified prognostic factors and to assess their prognostic value over established prognostic factors (replication and confirmation). We now explain these components in more detail.

### Exploration

Approaches to identify possible potential prognostic factors usually incorporate biological reasoning (“candidate” approach) and the hypothesised causal pathway from the onset of disease or condition to subsequent outcome. Although there are few, if any, conditions for which there is no information on prognostic factors, there is a growing role for hypothesis-free (“biology agnostic”) studies to discover previously unsuspected factors. Such studies do not focus on one (or a few) specific prognostic factors, but rather investigate many factors (for example, 10–20 psychosocial factors or millions of genetic variants) and their association with outcome. Although sometimes overlooked, simple clinical information and factors used to diagnose a disease or health condition may be used in exploration. The availability of new analytical technologies has supported recent rapid growth in the use of “omic” [42],[43] approaches to discover potential prognostic factors using DNA (genomics), RNA (expression products, transcriptomics) [5], proteins (proteomics), or metabolites (metabolomics).

### Replication and Confirmation

Once potential prognostic factors have been identified in one study, early replication in multiple independent studies is important, together with assessment of prognostic value over other factors. For example, a meta-analysis of individual participant data from six studies in traumatic brain injury showed that blood glucose has incremental prognostic value over established prognostic factors of age, motor score, and pupillary reactivity in relation to a poor outcome (a Glasgow outcome score of 1–3 at 6 months) (see Figure S1) [33].

### Illustrative Example

**Figure 3:**
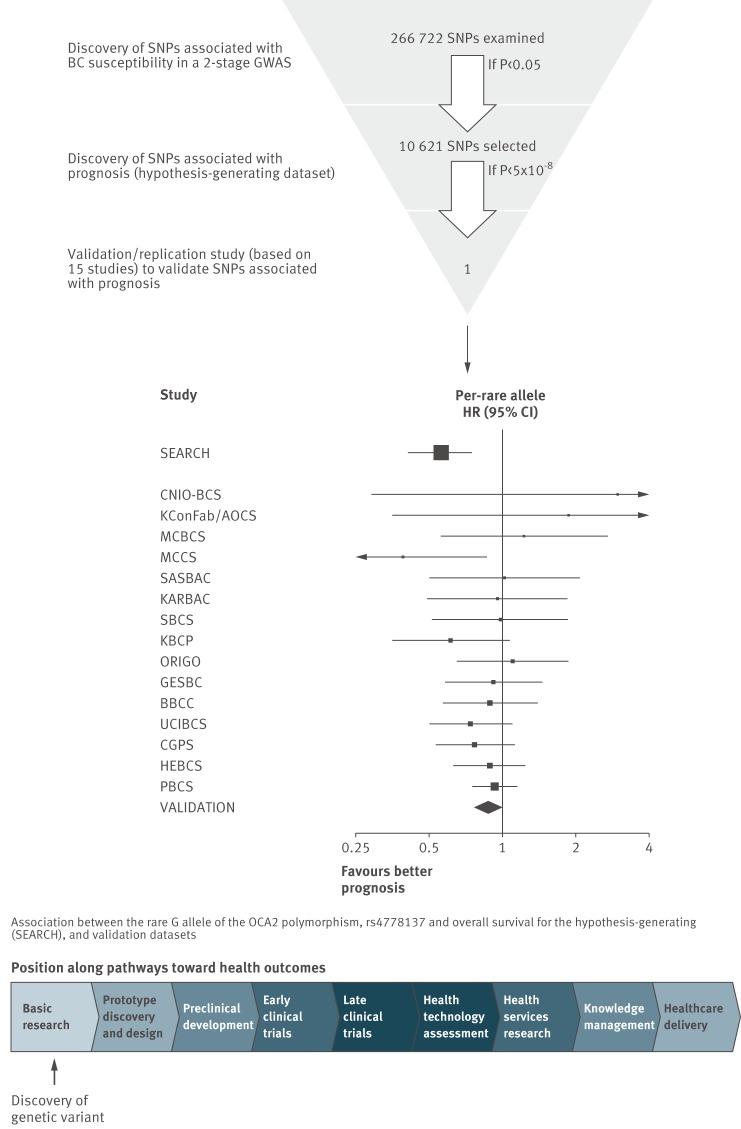
Discovery of prognostic factors: a genome-wide association study of survival among people with breast cancer, and replication in 15 studies (Mid-figure forest plot based on example plots in [4]. Path element adapted from Chart 7.1 in the Cooksey report (2006) http://bit.ly/Ro27rL (made available for use and re-use through the Open Government License).

The standard of research that has emerged in genome-wide association studies is that discovery and multiple replication are combined in the first publication (illustrated in Figure 3). An exploratory study, with no prior hypotheses about which genes would be prognostic, identified an association between a variant in the gene called OCA2 with survival among women with oestrogen receptor negative breast cancer; 15 additional studies were then synthesised immediately and successfully replicated this association [4]. An implication of this finding for clinical research is that, because of the biology of OCA2, this prognostic factor should be tested as a candidate for predicting differential treatment response to anthracycline drugs.

## Prognostic Factor Research: Recommendations for Improvement

Given the abundance of prognostic factor research, it is surprising that the methodology regarding the design, conduct, and analysis of prognostic factor studies is not well established [34],[41],[44]–[47]. Further, an increasing body of evidence has highlighted severe limitations [3],[10]–[12]. The studies are often poorly designed [41],[45], inappropriately analysed [44],[48], and poorly reported [28],[49],[50]. For example, in a review of prognostic factor studies in paediatric oncology, prognostic effect sizes (such as hazard ratios) and their confidence intervals could be extracted for only 35.5% of the prognostic factor assessments reported [51]. Replication of initial prognostic factor evidence is also poor. For example, a review of prognostic factors in neuroblastoma found that 130 different genetic and biological factors had been investigated in 211 published studies, with a median of one publication per factor. Publication bias and selective reporting of primary studies seems endemic [52]. For example, Kyzas et al evaluated 1575 articles on different prognostic factors for cancer, and staggeringly found that nearly all suggested significant findings, with 98.5% reporting statistically significant results or elaborating on non-significant trends [52].

These problems often result in confusion about the prognostic value of individual factors [12], and consequently genuine prognostic factors have a smaller impact than they ought to have on improving health outcomes. Box 1 illustrates, across a wide variety of clinical fields, the frustration of those attempting to conduct and draw inferences from systematic reviews and meta-analyses of published prognostic factor studies [4],[11],[53]–[60]. Clearly standards must be raised. Many of the recommendations highlighted across the PROGRESS series (see Table S1) are relevant. Here we make recommendations within five priority areas. Many of these are also relevant for the other types of prognosis research considered in our series.

**Box 1. Evidence from systematic reviews and overviews indicating that the quality of prognostic factor research needs to improve**

**General**

“As a consequence of the poor quality of research, prognostic markers may remain under investigation for many years after initial studies without any resolution of the uncertainty. Multiple separate and uncoordinated studies may actually delay the process of defining the role of prognostic markers” [53]

“The (prognostic factor) literature is probably cluttered with false-positive studies that would not have been submitted or published if the results had come out differently” [11]

**Bladder cancer**

“After 10 years of research, evidence is not sufficient to conclude whether changes in P53 act as markers of outcome in patients with bladder cancer…. That a decade of research on P53 and bladder cancer has not placed us in a better position to draw conclusions relevant to the clinical management of patients is frustrating” [54]

**Coronary disease**

“Multiple types of reporting bias, and publication bias, make the magnitude of any independent association between CRP and prognosis among patients with stable coronary disease sufficiently uncertain that no clinical practice recommendations can be made” [55]

**Musculoskeletal disorders: low back pain**

“We observed the potential impact of different methods on the results of systematic reviews in the area of low back pain prognosis. This emphasizes the need for cautious interpretation and careful attention to methods and transparent reporting in future reviews. There is an immediate need for methodological work in the area of prognosis systematic reviews to investigate potential biases” [60]

**Acute orthopaedic trauma**

“There was limited evidence for the role of any factor as a predictor of return to work.… Due to the lack of factors considered in more than one cohort, the results of this review are inconclusive. The review highlights the need for more prospective studies that are methodologically rigorous, have larger sample sizes and consider a comprehensive range of factors” [56]

**Whiplash**

“Data regarding the prognostic factors associated with poor recovery were difficult to interpret due to heterogeneity of the techniques used to assess such associations and the way in which they are reported. There was also wide variation in the measurement of outcome and the use of validated measures would improve interpretability and comparability of future studies” [57]

**Osteosarcoma**

“93 papers were studied in depth…. Only 7 papers were of sufficient quality to analyze.… Because of heterogeneity of the studies, pooling results is hardly possible. There is a need for standardization of studies and report” [58]

**Peptic ulcer perforation**

“Fifty prognostic studies with 37 prognostic factors comprising a total of 29,782 patients were included in the review. The overall methodological quality was acceptable, yet only two-thirds of the studies provided confounder adjusted estimate” [59]

### Planning, Design, and Analysis

Researchers and funders should develop a clearer understanding of the progression of research evidence about prognostic factors: from initial discovery, through to replicable evidence of prognostic ability, and application (or being discarded, if appropriate) (recommendation 9 in Table S1). Study objectives should be presented in the context of existing evidence. Guidelines for those planning and undertaking a prognostic factor study have been suggested [3],[41],[45] and should be used to ensure higher standards of study quality, design, and analysis than is currently observed and to emulate the standards set by randomised trials (recommendation 10) [61]. These should include the need for study registration, a published protocol, ideally a prospective approach, and an appropriate statistical analysis plan.

Registration of protocols in a publically accessible register (such as clinicaltrials.gov) would make others aware of ongoing research, encourage the pre-specification of objectives and factors of interest, and reduce publication and selective reporting biases (recommendations 11 and 12). A prospective rather than retrospective design is preferable (recommendation 10) as it enables clear inclusion criteria, more complete baseline and follow-up data, and greater standardisation of diagnostic and therapeutic procedures, and ensures the primary factors and outcomes are specified in advance, reducing the potential for data dredging and thus type I errors. This is especially important for larger studies aiming to replicate earlier exploratory prognostic factor findings, and these should incorporate a suitable sample size calculation to ensure adequate power to detect a prognostic effect, if it exists. Statistical analysis methods can be substantially improved [44] by analysing continuous factors on their continuous scale, thereby avoiding the use of arbitrary cut-points to categorise them [48], by considering non-linear relationships, and by including multivariable analyses that assess a factor's prognostic value over existing prognostic factors [44] (recommendation 13).

For many diseases and health conditions there is a lack of clinical cohorts to evaluate prognostic factors appropriately. New clinical cohorts should be established in which consenting individuals with specified health related condition(s) (including diagnosed disease, and symptoms) are phenotyped and have multiple baseline characteristics measured, are placed within a biorepository (if appropriate), and are followed up with their multiple health outcomes recorded (and linked to and from other databases as necessary). Research funders should help establish new investigator-led clinical cohorts that meet the criteria which we set out; currently healthy population cohorts tend to predominate (recommendation 14).

### Nomenclature and Quality of Reporting

Prognostic factor studies must also improve their transparency of reporting [49] in order to help promote new prognostic factors, resolve ongoing debate over the prognostic value of existing factors, distinguish good quality research from low quality research, facilitate systematic reviews and meta-analyses in the subject [46],[62], and ultimately help decision makers use prognostic factor evidence (recommendation 15). The REMARK reporting guidelines [63] provide recommendations for prognostic factor studies in oncology, but most of these recommendations are generalisable to non-cancer diseases.

Another barrier to interpreting prognostic factor research is the inconsistent nomenclature used both within and across disease specialties. For example, prognostic factors are alternatively known as prognostic variables, prognostic (bio)markers, prognostic indicators, prognostic determinants, predictors, or molecular markers, among others. Prognosis research publications should use standard terms and nomenclature in order for different laboratory, clinic, and population disciplines to interact, and for research findings to be interpreted appropriately and consistently by clinicians and patients (recommendation 16).

### Replication, Data Sharing, and Evidence Synthesis

As there is a need for initial evidence of a prognostic factor to be shown as consistent in subsequent studies, researchers must support systematic reviews and evidence synthesis of prognostic factor studies. In particular, we strongly support calls for authors to facilitate meta-analysis of individual participant data from prognostic factor studies through data sharing initiatives (recommendation 17) [3],[49]. Such an analysis uses original source data at the participant level and has many advantages over a meta-analysis of summary results from the literature [64]. In particular, one can derive desired prognostic factor results directly, independent of study reporting and significance, and analyse continuous factors more appropriately [37]. Meta-analyses of individual participant data are achievable for prognostic factors: for example, collaborators united to provide individual participant data from 11 studies of traumatic brain injury to form a prognostic factor database including 9205 patients [32]. This should be encouraged in other specialties, and ideally meta-analyses of individual participant data should be prospectively planned [3] (a design used for over a decade in epidemiology [65]) to minimise heterogeneity between studies (such as in factors assessed, methods of measurement, and inclusion criteria) and unavailability of data [66].

### Impact of Research Findings

Prognostic factor studies need to improve in providing a clear message about the implications of their findings for clinical practice, randomised trials, or further research. For example, prognosis researchers should consider if further research is needed to confirm that a factor has prognostic value. If there is consistent evidence of prognostic value, then what are the potential uses of the prognostic factor within healthcare research and clinical decision making? Are there any implications for the use of known, or the development of novel, interventions? Such considerations should be made in order to help move prognostic factor research findings into the different translational pathways toward improving clinical outcomes (recommendation 18).

Many of the uses of prognostic factors—such as informing diagnosis, tailoring treatment decisions, and monitoring patients—constitute a health technology for clinical practice. However, the potential impact of implementing prognostic factors—in terms of costs, outcomes, and broader healthcare impacts—are rarely evaluated (unlike with drugs and interventions), and this should be addressed (recommendation 19). Indeed, the nature and extent of evidence required to do this must also be clarified. This echoes recent calls for randomised trials [67] or decision modelling techniques [24],[68] to investigate the added value of prognostic factors in clinical practice.

## Conclusion

Prognostic factors have the potential to play an important role in pathways towards improved health, including clinical practice, healthcare research, and the development, evaluation, and targeting of interventions. Improvements in the design, conduct, analysis, and reporting of prognostic factor research are crucial to enable more reliable prognostic factor evidence that can be used in these pathways and ultimately help improve patient outcomes.

## Supporting Information

Figure S1 **Blood glucose as a prognostic factor in traumatic brain injury (drawn using data from** **[32]**,**[33]** **).** The forest plots shows two random-effects meta-analyses of individual participant data from 6 studies, aiming to establish whether glucose is a prognostic factor of unfavourable six month outcome (defined by a Glasgow Outcome Score of 1, 2 or 3) in patients with traumatic brain injury. The meta-analysis in (A) confirms that glucose is a prognostic factor, as the odds of the outcome increase as glucose levels increase (odds ratio >1). Further, the meta-analysis in (B) shows that glucose is an ‘independent’ prognostic factor, as its prognostic value largely remains even after adjusting for the other prognostic factors of age, motor score and pupillary reactivity.
(DOC)

Table S1 **Recommendations of PROGRESS (PROGnosis RESearch Strategy).**
(DOC)
