# Supplementary material for: Prognosis Research Strategy (PROGRESS) 2: Prognostic Factor Research
Source: PLoS Med. 2013 Feb 5;10(2):e1001380. doi: 10.1371/journal.pmed.1001380 (PMC3564757; doi:10.1371/journal.pmed.1001380)
Supplement: Figure S1 — Blood glucose as a prognostic factor in traumatic brain injury (drawn using data from [32] , [33] ). The forest plots shows two random-effects meta-analyses of individual participant data from 6 studies, aiming to establish whether glucose is a prognostic factor of unfavourable six month outcome (defined by a Glasgow Outcome Score of 1, 2 or 3) in patients with traumatic brain injury. The meta-analysis in (A) confirms that glucose is a prognostic factor, as the odds of the outcome increase as glucose levels increase (odds ratio >1). Further, the meta-analysis in (B) shows that glucose is an ‘independent’ prognostic factor, as its prognostic value largely remains even after adjusting for the other prognostic factors of age, motor score and pupillary reactivity. (DOC) [file pmed.1001380.s001.doc]

. (1.05, 1.24)

. (1.03, 1.22)

with estimated prediction interval

with estimated prediction interval

.

.

.

.

(A) Analysis unadjusted for other factors

1

2

3

4

5

6

Subtotal (I-squared = 49.2%, p = 0.080)

(B) Analysis adjusted for age, motor score, & pupils

1

2

3

4

5

6

Subtotal (I-squared = 48.2%, p = 0.086)

trial

1.13 (1.09, 1.18)

1.18 (1.13, 1.23)

1.20 (1.14, 1.27)

1.12 (1.08, 1.16)

1.08 (1.00, 1.16)

1.12 (0.98, 1.28)

1.14 (1.11, 1.18)

1.11 (1.07, 1.15)

1.14 (1.09, 1.19)

1.21 (1.14, 1.28)

1.10 (1.06, 1.14)

1.06 (0.97, 1.15)

1.11 (0.96, 1.28)

1.12 (1.09, 1.16)

ratio (95% CI)

odds

1.13 (1.09, 1.18)

1.18 (1.13, 1.23)

1.20 (1.14, 1.27)

1.12 (1.08, 1.16)

1.08 (1.00, 1.16)

1.12 (0.98, 1.28)

1.14 (1.11, 1.18)

1.11 (1.07, 1.15)

1.14 (1.09, 1.19)

1.21 (1.14, 1.28)

1.10 (1.06, 1.14)

1.06 (0.97, 1.15)

1.11 (0.96, 1.28)

1.12 (1.09, 1.16)

ratio (95% CI)

odds

1

.9

1

1.1

1.2

1.3

1.4

odds ratio

NB The odds ratio gives the ratio of odds for two patients who differ in glucose by 1 mMol/l.

The prediction interval gives a 95% interval for the true prognostic effect of glucose in an individual study setting.
